# Efficacy of Combined *Hericium erinaceus* Mycelium and Undenatured Type II Collagen in Reducing Osteoarthritis Progression in a Preclinical Animal Model

**DOI:** 10.7150/ijms.126220

**Published:** 2026-01-23

**Authors:** Kun-Tsan Lee, Chin-Jung Hsu, Li-Chai Chen, Li-Ya Lee, Wan-Ping Chen, Yu-Wen Chen, Chin-Chu Chen, Yen-You Lin, Tzu-Ching Chang, Chen-Ming Su, Chih-Hsin Tang

**Affiliations:** 1 Department of Post-Baccalaureate Medicine, National Chung-Hsing University, Taichung, Taiwan.; 2 Department of Orthopedics, Taichung Veterans General Hospital, Taichung, Taiwan.; 3 School of Chinese Medicine, China Medical University, Taichung, Taiwan.; 4 Department of Orthopedic Surgery, China Medical University Hospital, Taichung, Taiwan.; 5 Department of Pharmacy, Tajen University, Pingtung, Taiwan.; 6 Biotech Research Institute, Grape King Bio Ltd., Taoyuan City, Taiwan.; 7 Institute of Food Science and Technology, National Taiwan University, Taipei City, Taiwan.; 8 Translational Medicine Center, Shin Kong Wu Ho-Su Memorial Hospital, Taipei, Taiwan.; 9 Department of Pharmacology, School of Medicine, China Medical University, Taichung, Taiwan.; 10 Department of Sports Medicine, College of Health Care, China Medical University, Taichung, Taiwan.; 11 Chinese Medicine Research Center, China Medical University, Taichung, Taiwan.; 12 Department of Medical Laboratory Science and Biotechnology, College of Medical and Health Science, Asia University, Taichung, Taiwan.

**Keywords:** osteoarthritis, *Hericium erinaceus*, UC-II, combination treatment

## Abstract

Osteoarthritis (OA) is a condition linked with aging that impacts joints and leads to functional disability. *Hericium erinaceus*, a large edible mushroom widely consumed in Asian countries, is recognized as a functional food and has been reported as a beneficial supplement for OA management. Undenatured type II collagen (UC-II), a new nutraceutical ingredient, has garnered significant interest for its potential in OA treatment. This study investigated whether *H. erinaceus* mycelium (HEM) and UC-II together are more effective at preventing the advancement of OA. HEM and UC-II reduce bone pain and the development of OA associated with anterior cruciate ligament transaction. Through the reduction of pro-inflammatory cytokines IL-1β and TNF-α, as well as the chondrolytic factors MMP-3, MMP-13, and ADAMTS5, HEM and UC-II inhibited the degradation of aggrecan and COL2A1. This action resulted in a blockade of cartilage breakdown and bone loss. The combination of HEM and UC-II also prevented OA progression. These findings provide evidence for using HEM and UC-II for OA therapy.

## Introduction

As a result of medical progress and the rise in life expectancy, degenerative disorders have emerged as some of the highest widespread health issues, with osteoarthritis (OA) being one of the most common. The Global Burden of Disease report indicated that around 528 million people globally were affected by OA, with prevalence rising by 114.5% over a ten-year period 1. OA is characterized by pathological features such as subchondral bone sclerosis, cartilage degradation, and inflammation of the synovial tissue. These features are often irreversible at the time of diagnosis and lead to joint pain and stiffness 2, 3. At present, OA cannot be cured, and treatment options are restricted to slowing the advancement of the disease or easing pain.

Chronic inflammation of the synovial tissues is strongly linked to joint pain, structural damage, and the release of synovial fluid, which is crucial in promoting inflammation and tissue destruction in OA 4, 5. The upregulated levels of proinflammatory cytokines like IL-1β and TNF-α, together with synovium-related factors such as MMP-3, MMP-13, and ADAMTS5, show a significant correlation with the severity of knee OA and may be linked to the progression of OA 6-9. The rising levels of inflammatory cytokines and degradative enzymes lead to the breakdown of various components of the cartilage extracellular matrix, including aggrecan and collagen II 10, 11. According to pertinent research, methods that counteract inflammation could serve as a possible treatment for OA 4, 12.

*Hericium erinaceus*, a large edible mushroom widely eaten in Asian countries, is recognized as a dietary supplement or functional food 13, 14. *H. erinaceus* contains a wealth of bioactive substances, such as ketones, polysaccharides, and glycoproteins 26. Moreover, the fruiting bodies, mycelium, and bioactive pure compounds of *H. erinaceus* demonstrate various medicinal properties, such as anti-inflammatory, anti-cancer, and neuroprotective functions 13, 15, 16. *H. erinaceus* mycelium (HEM) demonstrated anti-inflammatory and chondroprotective effects *in vivo* 17. Clinical studies of collagen supplementation, like undenatured type II collagen (UC-II), in OA patients have yielded promising outcomes, with multiple studies showing enhancements in knee function and pain relief 18, 19. By promoting the production of extracellular matrix macromolecules, UC-II may stimulate cartilage regeneration 20. This study investigated whether HEM and UC-II together are more effective at preventing the advancement of OA. In this study, we discovered that both HEM and UC-II prevent the onset of OA induced by anterior cruciate ligament transection (ACLT)* in vivo*. Moreover, the synergy of HEM and UC-II also shows prevented OA progression. These findings provide evidence for using HEM and UC-II for OA therapy.

## Materials and Methods

### Materials

Aggrecan (ab3778) antibody was obtained from Abcam (Cambridge, UK). MMP-3 (SC-21732) and MMP-13 (SC-30073) antibodies were obtained from Santa Cruz Biotechnology (Dallas, TX, USA). TNF-α (A11534), COL2A1 (A1560) and ADAMTS5 (A2836) antibodies were obtained from ABclonal, Inc. (Woburn, MA, USA). IL-1β (MAB601) antibody was obtained from R&D Systems, Inc. (Minneapolis, MN, USA).

### Preparation of HEM

HEM (No. 35669) was obtained from the Bioresource Collection and Research Center (Hsinchu, Taiwan). The HEM powder was prepared according to our previous report 17. UC-II was purchased for Lonza (Basel, Switzerland).

### ACLT animal model

Male Sprague Dawley (SD) rats, aged 8 weeks and weighing between 300 and 350 g, were acquired from the National Laboratory Animal Center in Taipei, Taiwan. They were randomly assigned to one of five groups: sham surgery (controls), ACLT only, ACLT with HEM (100 mg/kg), ACLT with UC-II (4 mg/kg) and ACLT with HEM plus UC-II. The ACLT operation was conducted following the procedure described in our earlier documents 21, 22.

In line with our earlier protocols 23, 24, the weight-bearing incapacitance test was investigated weekly to assess spontaneous pain following ACLT, based on variations in dynamic weight bearing between the resting right and left hind limbs.

### μ-CT measurements

Following 6 weeks of application, the rats were sacrificed. Their intact right knee joints were then scanned with a SkyScan 2211 μ-CT system (Bruker; Kontich, Belgium) and analyzed using CTAn software, in line with our earlier protocols 23, 25.

### Histological analysis

As reported earlier 26, 27, histopathological changes in OA tissue were examined using Hematoxylin and eosin (H&E) and Safranin-O/Fast Green staining under an optical microscope. Knee joint tissues were fixed in 4% formaldehyde and decalcified using 10% EDTA in PBS for 14 days. This was followed by dehydration with ethanol. The specimens were subsequently embedded in paraffin blocks and sliced into 5 µm thick sections for histological staining. The Osteoarthritis Research Society International (OARSI) histopathology assessment system 28 was utilized to evaluate structural changes in the cartilage of the central weight-bearing area of the medial tibial plateau. This system incorporates grading and staging scores to indicate the depth of lesions and the severity of OA, respectively.

### Immunohistochemistry (IHC) staining

As detailed in reference 29, the analysis of immunohistochemistry was performed using the Leica Novolink Polymer Detection system (Leica Biosystems Inc, IL, USA). Tissue sections were briefly applied with 3% hydrogen peroxide and then treated with 3% bovine serum albumin in PBS. The sections were applied with primary antibodies at 4°C overnight, followed by a 1-hour incubation with a peroxidase-conjugated secondary antibody at room temperature and staining with diaminobenzidine substrate.

### Statistical analysis

Statistical analyses for quantified results were conducted using GraphPad Prism 5.0 software. Data are presented as the mean ± standard deviation (S.D.). The paired sample t-test and One-way ANOVA followed by Bonferroni post hoc testing was used to compare results from two groups and from more than two groups, respectively. Statistical significance was determined by a *p*-value of less than 0.05 in all cases.

## Results

### HEM and UC-II do not affect the body weight growth curve

**Figure 1:**
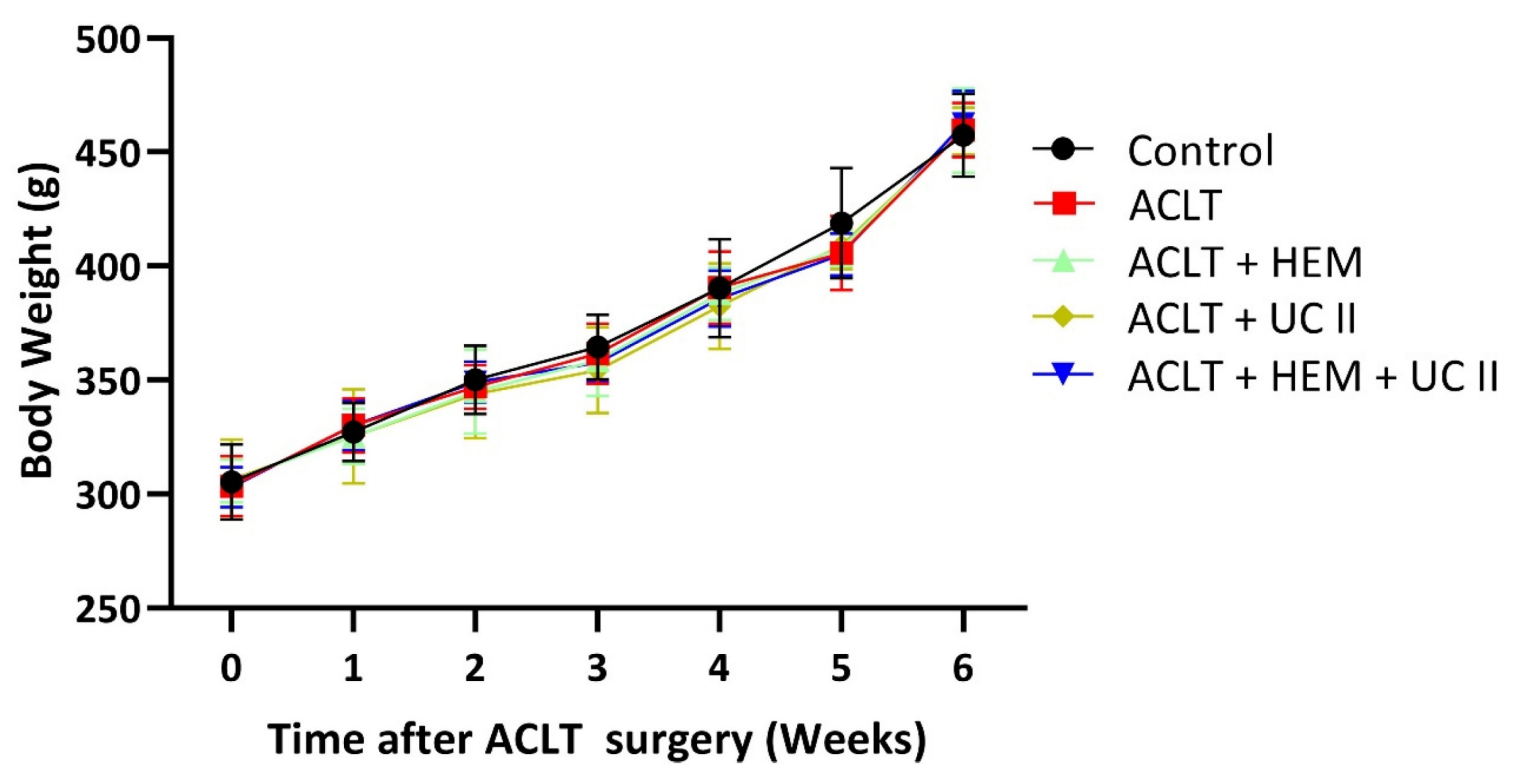
** Increase in body weight throughout the experimental phase.** Throughout the course of the experiment, body weight was measured.

We employed a rat model of ACLT-induced knee arthritis to examine the protective effects of HEM and UC-II. Pain behavior assessments and histological analyses were carried out to investigate the underlying mechanisms. The day prior to surgery, the rats' body weights were documented, and this process continued weekly until the rats were sacrificed. Throughout the duration of the experiment, all groups exhibited a gradual increase in body weight, and no significant differences were found among the groups (Figure 1). Our findings suggest that neither HEM, UC-II, nor their combination exhibits no toxic side effects affecting body weight.

### HEM and UC-II ameliorate OA pain

**Figure 2:**
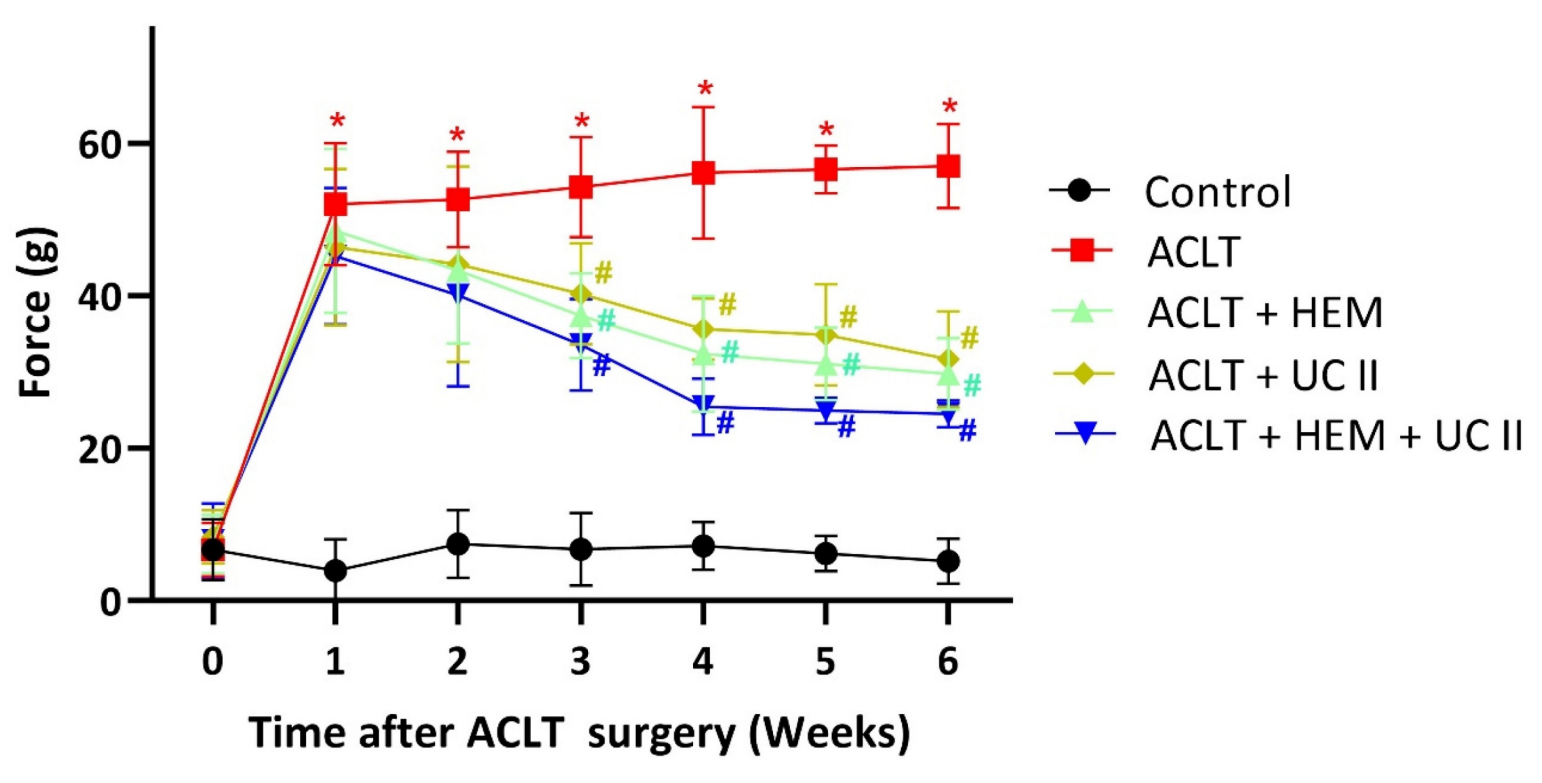
** HEM and UC-II decelerate ACLT-induced bone pain.** Every week, weight-bearing behavioral testing was conducted to assess deficits in weight-bearing forces. * *p*<0.05 compared with the control group; # *p*<0.05 compared with the ACLT-only group.

The static weight-bearing incapacitance test was used to investigate the pain behavior of the rats. During the first week after surgery, all groups demonstrated a severe asymmetrical weight-bearing posture (Figure 2). In the ACLT rats, this serious asymmetry intensified over the course of the experiment. On the other hand, the ACLT+HEM and ACLT+UC-II groups noted significant enhancements in pain-related behavior (Figure 2). The combination of HEM and UC-II exhibits greater efficacy (Figure 2). These results suggest that HEM, UC-II, and their combination effectively alleviate OA-related pain.

### HEM and UC-II protect against ACLT-induced osseous and cartilage damage in an ACLT-induced OA model

**Figure 3:**
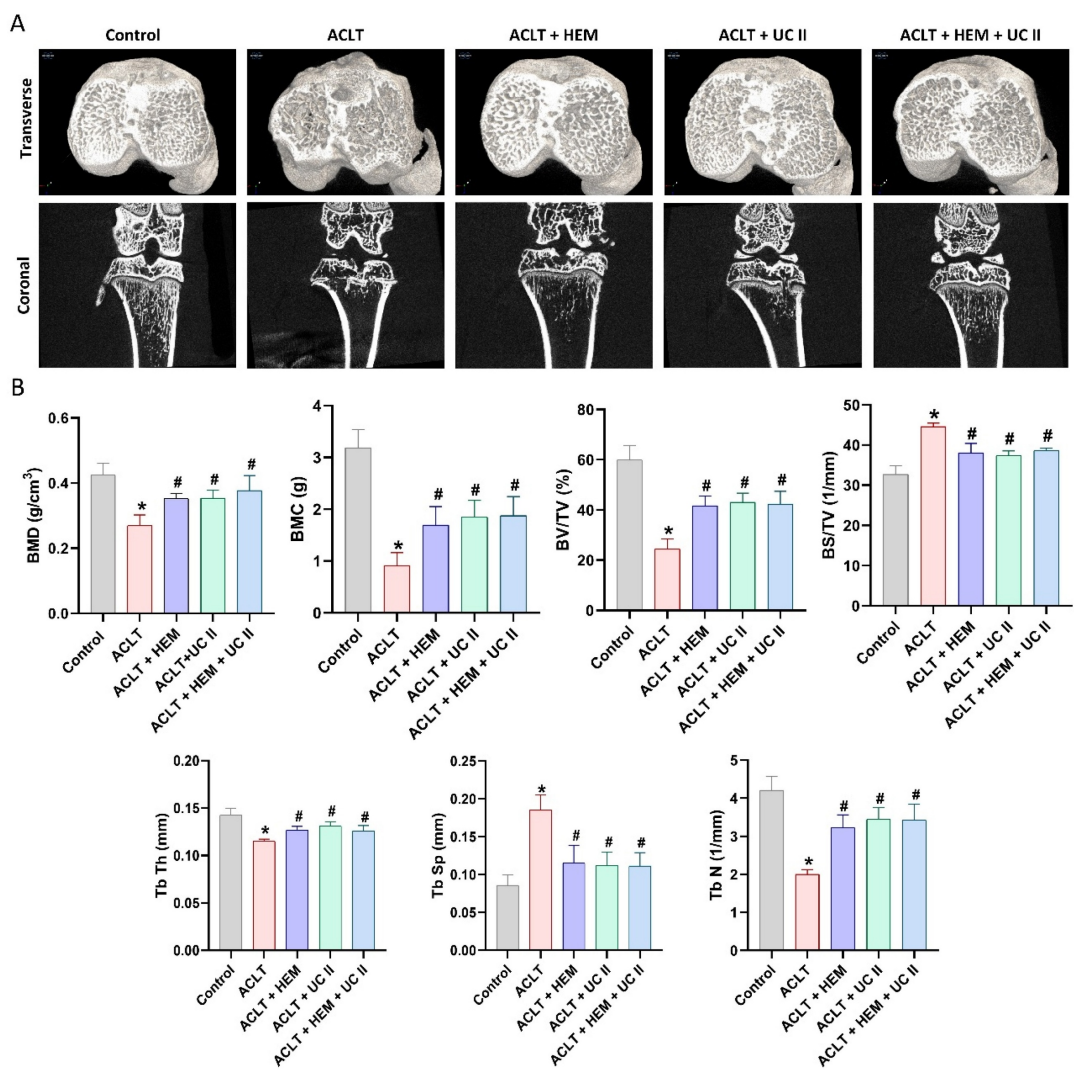
** HEM and UC-II ameliorate osseous damage in the ACLT-induced OA knee joint.** (A) Representative micro-CT images from knee subchondral bone. (B) Quantitative analyses of BMD, BMC, BV/TV, BS/TV, Tb.Th, Tb.N, and Tb.Sp. * *p*<0.05 compared with the control group; # *p*<0.05 compared with the ACLT-only group.

Six weeks after ACLT surgery, μ-CT analysis was conducted to evaluate changes in trabecular microarchitecture. In ACLT rats, significant bone damage was observed compared to controls, confirming the OA lesion resulting from ACLT surgery (Figure 3). Quantitative analysis indicating the decrease of bone mineral density (BMD), bone mineral content (BMC), bone volume/tissue volume ratio (BV/TV), bone surface to tissue volume ratio (BS/TV), trabecular thickness (Tb.Th) and trabecular number (Tb.N) together with a rise in trabecular separation (Tb.Sp) in ACLT rats (Figure 3). Furthermore, significant improvements in bone architecture were found in rats treated with HEM, UC-II, or their combination compared to the ACLT group (Figure 3).

**Figure 4:**
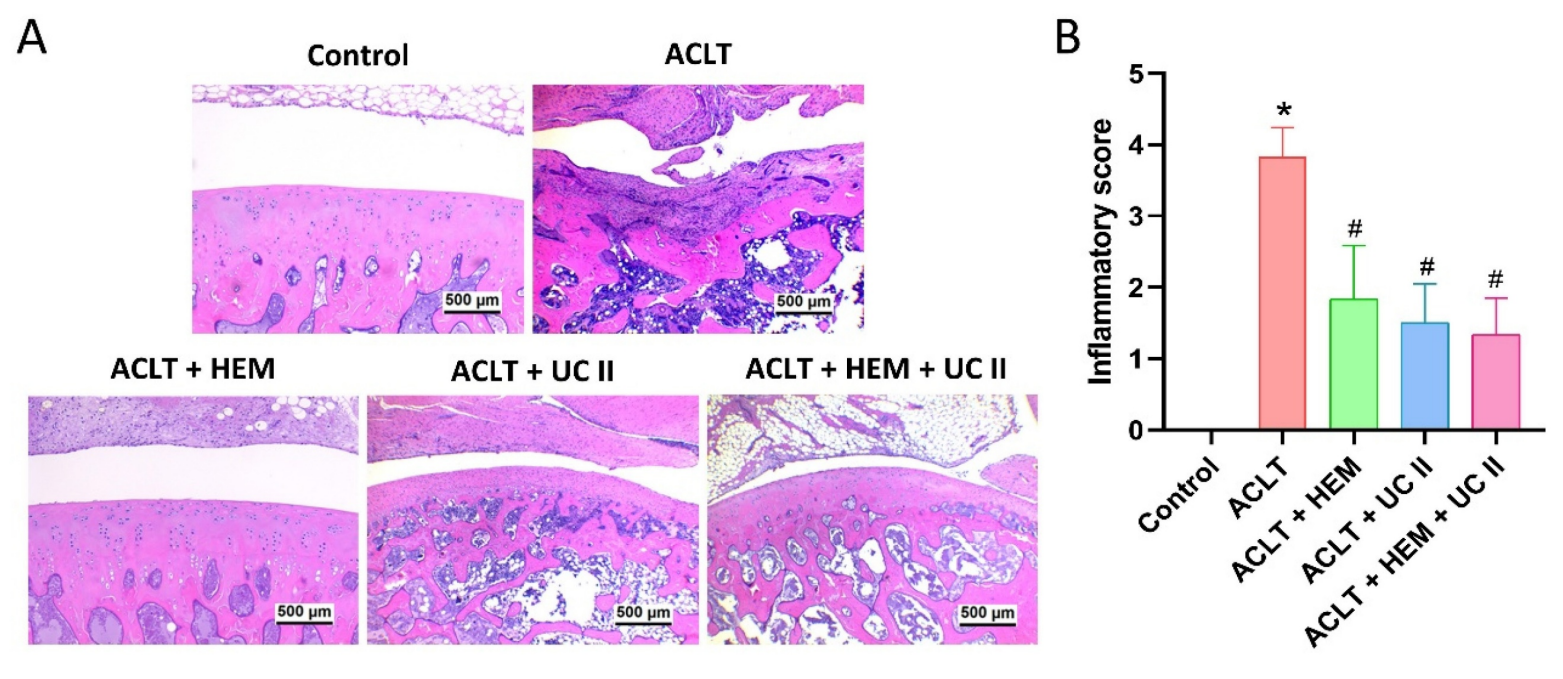
** HEM and UC-II block ACLT-induced synovial inflammation and cartilage degradation.** (A) Histological sections from knees stained with H&E. (B) Quantitative analyses of synovium scores. Scale bar = 500 μm. * *p*<0.05 compared with the control group; # *p*<0.05 compared with the ACLT-only group.

**Figure 5:**
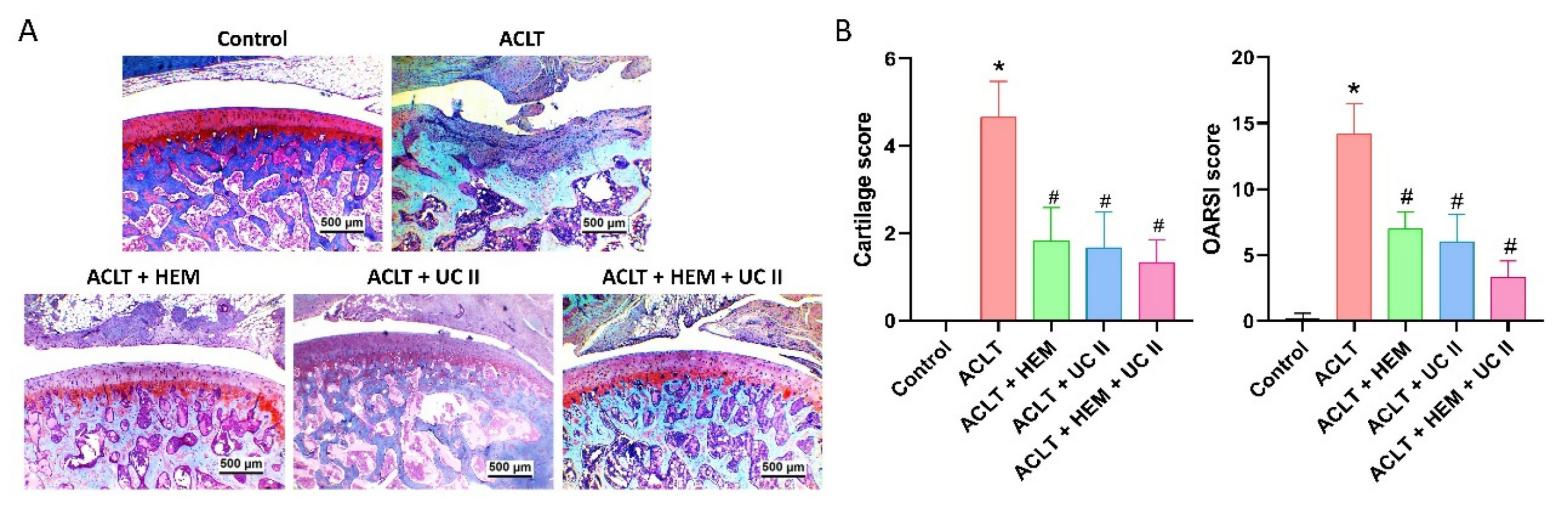
** HEM and UC-II block ACLT-induced cartilage breakdown.** (A) Histological sections from knees stained with Safranin-O. (B) Quantitative analyses of OARSI and cartilage scores. Scale bar = 500 μm. * *p*<0.05 compared with the control group; # *p*<0.05 compared with the ACLT-only group.

Histological analysis with H&E and Safranin-O/Fast Green staining found degradation of articular cartilage and hyperplasia of the synovial lining in the ACLT knee groups (Figure 4&5). The quantification of inflammation, OARSI scores, and cartilage scores showed that the ACLT+HEM, ACLT+UC-II, and ACLT+HEM+UC-II groups had lower pathological changes in cartilage tissue and less synovial tissue hyperplasia compared to the ACLT group (Figure 4&5).

### HEM and UC-II suppress proinflammatory cytokine production and cartilage degradation

**Figure 6:**
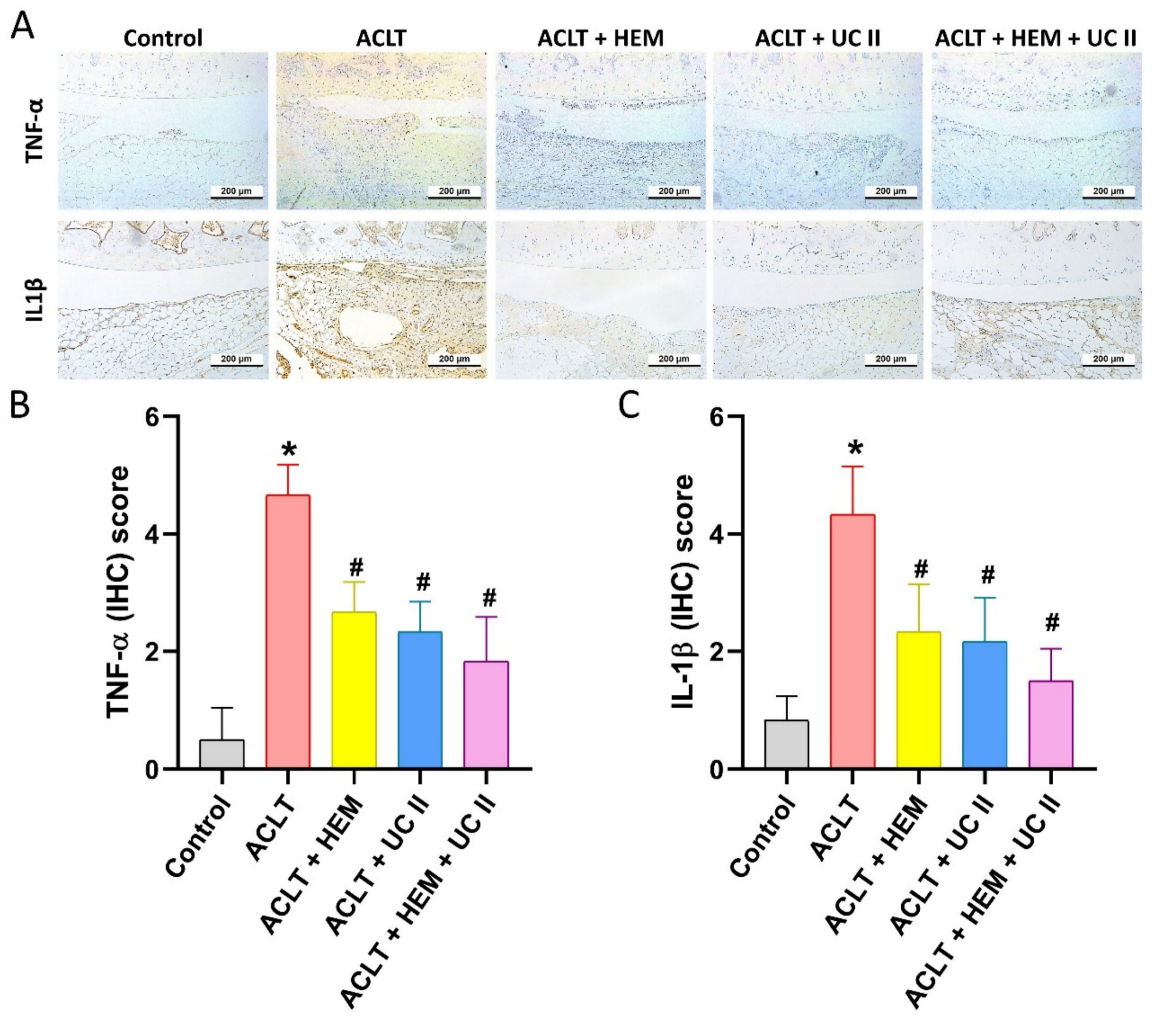
** HEM and UC-II diminish the induction of IL-1β and TNF-α in ACLT-induced OA articular cartilage.** Immuno-histochemistry analysis and scoring of IL-1β (A, B) and TNF-α (A, C) in rat knee joint cartilage. Scale bar = 200 μm. * *p*<0.05 compared with the control group; # *p*<0.05 compared with the ACLT-only group.

**Figure 7:**
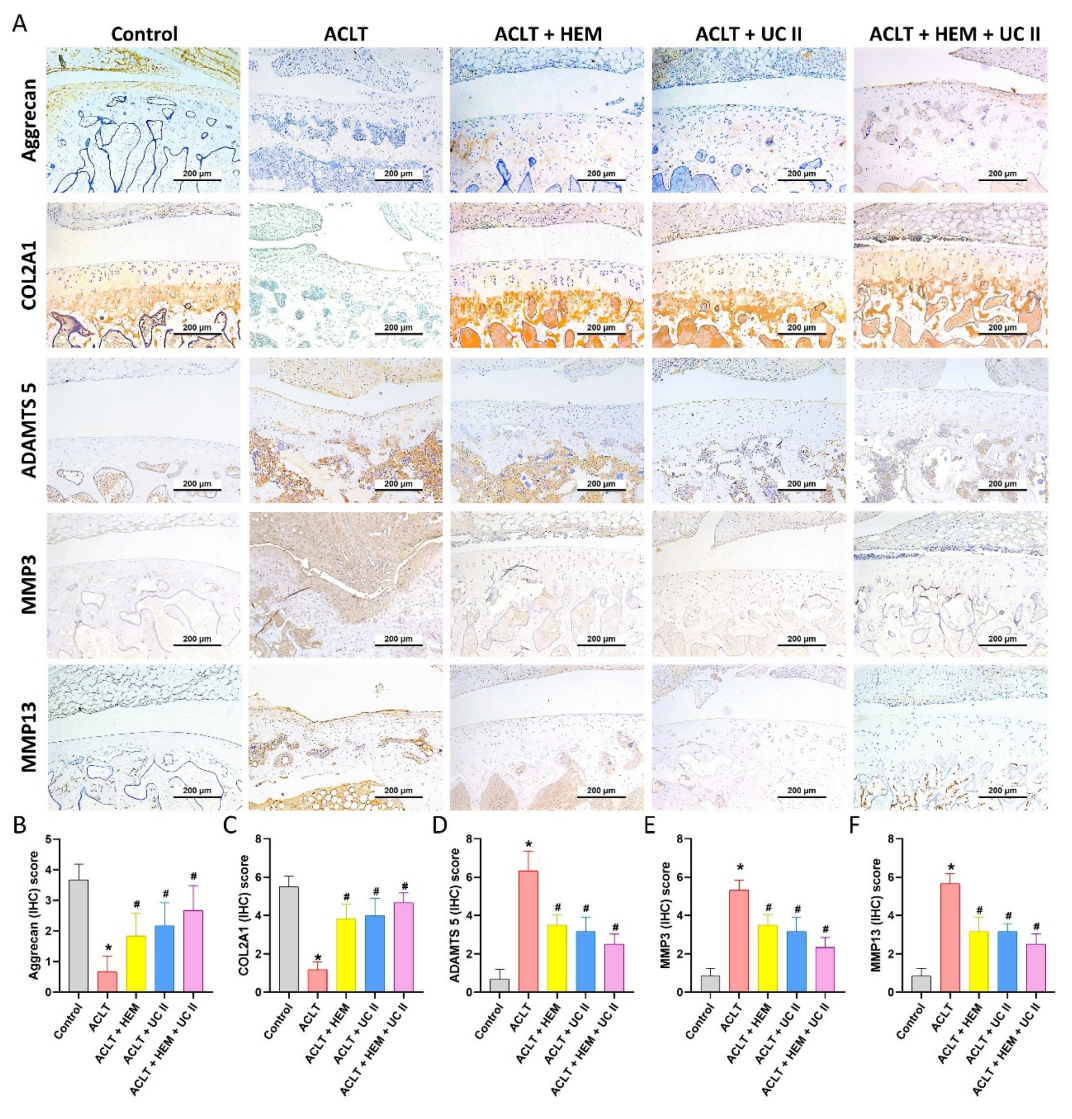
** HEM and UC-II reserve the expression of aggrecan and COL2A1 accompanying with suppression of MMP-3, MMP-13 and ADAMTS5 in ACLT-induced OA articular cartilage.** (A) Immuno-histochemistry analysis MMP3, MMP-13, ADAMTS5, aggrecan and COL2A1 in rat knee joint cartilage. (B-F) Scoring of the immunosignals of MMP3, MMP-13, ADAMTS5, aggrecan and COL2A1. Scale bar = 200 μm. * *p*<0.05 compared with the control group; # *p*<0.05 compared with the ACLT-only group.

The IHC analysis showed a significant elevate in the production of IL-1β and TNF-α in the synovial tissue of the ACLT group, suggesting an escalation of inflammatory activity. As depicted in Figure 6, this elevation was significantly diminished in the ACLT+HEM, ACLT+UC-II, and ACLT+HEM+UC-II groups. IHC staining of MMP-3, MMP-13, ADAMTS5, aggrecan, and type II collagen alpha1 chain (COL2A1), which forms the basis for articular cartilage, was used to conduct further assessment of cartilage metabolism. Compared to the ACLT group, the ACLT+HEM, ACLT+UC-II, and ACLT+HEM+UC-II groups showed reduced levels of MMP-3, MMP-13, and ADAMTS5, along with augmented levels of aggrecan and COL2A1 (Figure 7). The combination of HEM and UC-II also has a protective role in maintaining cartilage integrity in the ACLT-induced OA model.

## Discussion

For thousands of years, natural products have been employed to treat human ailments, and *H. erinaceus* is a prominent element of traditional Chinese medicine 30, 31. The components of *H. erinaceus* have been studied, and their effects on various body systems, especially the nervous system 32, 33, have been recorded. Many bioactive components, including nutritional components, secondary metabolites and polysaccharides, are found in *H. erinaceus* 34. We previously found that HEM exhibits anti-inflammatory and chondroprotective functions in an OA model 17. Here, our results also confirm similar effects, demonstrating that HEM reduces OA-related pain and progression in an ACLT-induced OA model. HEM inhibits inflammatory cytokine expression and cartilage degradation *in vivo*. Additionally, we provide evidence that the combination of HEM and UC-II also has therapeutic potential for OA.

UC-II, a new nutraceutical ingredient, has garnered significant interest for its potential in OA treatment. UC-II is a new form of undenatured type II collagen sourced from the cartilage of chicken sternums. Compared to the chondroitin sulfate supplements and glucosamine typically used in joint health investigations, UC-II was found to be more effective 35. Through its oral tolerance mechanism, UC-II blocks inflammatory T-cell activations and enhances T-regulatory cells, thereby potentially reducing cartilage damage 36. Here, we used an ACLT-induced OA model and observed similar effects. UC-II administration reduces OA-related pain, as demonstrated by weight-bearing testing. μCT results reveal that UC-II prevents bone damage and breakdown. Importantly, UC-II diminishes cartilage degradation by inhibiting proinflammatory cytokine production and cartilage-metabolizing enzyme expression. These results provide evidence that UC-II is an effective supplement for OA management. Our findings also demonstrate that the combination of HEM and UC-II also prevents OA progression.

OA is a long-lasting inflammatory condition that impacts pain behavior, synovial inflammation, and cartilage breakdown 37, 38. Cytokines that promote inflammation, like IL-1β and TNF-α, are significant in the advancement of OA, leading to joint pain, heightened inflammatory responses, and disruptions in chondrocyte metabolism in OA 12. Furthermore, clinical data reported in earlier studies demonstrated that OA patients exhibit significantly elevated levels of IL-1β and TNF-α on synovial tissue and in serum 21, 39. During pre-clinical trials, IL-1β and TNF-α are significant targets for identifying effective therapeutic strategies for OA. Our OA model induced by ACLT showed that ACLT surgery replicates clinical characteristics, leading to heightened production of IL-1β and TNF-α in cartilage and synovial tissue. The administration of HEM and UC-II evidently led to a downregulation in the production of IL-1β and TNF-α in cartilage as well as synovial tissues. The combination of HEM with UC-II slightly enhances HEM's anti-inflammatory properties, indicating that the anti-OA effects of their combination are due to its ability to block IL-1β and TNF-α synthesis.

The gel-like matrix of cartilage is mainly composed of the proteoglycan aggrecan and collagen. The matrix's primary component, COL2, constructs a fibrous network foundation, while proteoglycans attract water fractions to create a gel that maintains the cartilage's inflated and resilient characteristics 40. The cartilage matrix, which houses chondrocytes, supports maintain their stability and a balanced metabolism 41. Cartilage-related disorders such as articular degeneration and joint inflammation occur when chondrocytes are unable to maintain metabolic homeostasis within the cartilage matrix 42, 43. Here, we found that ACLT reduced the expression of aggrecan and COL2A1 while increasing the expression of the chondrolytic factors MMP-3, MMP-13, and ADAMTS5. HEM, UC-II, or their combination inhibits OA progression by suppressing chondrolytic factor expression, thereby restoring chondroprotective properties.

The limitations of the current study should be discussed. Although our results demonstrated that both HEM and UC-II, when administered individually, can prevent the onset of OA induced by ACLT *in vivo*, some outcomes indicated that the combination of HEM and UC-II was more effective than either HEM or UC-II alone. However, only a single dose of each compound was tested in this study. Future studies exploring combinations with different doses may yield even better results.

To sum up, our findings demonstrate that HEM and UC-II reduce bone pain and the development of OA associated with ACLT. Through the reduction of pro-inflammatory cytokines IL-1β and TNF-α, as well as the chondrolytic factors MMP-3, MMP-13, and ADAMTS5, HEM and UC-II inhibited the degradation of aggrecan and COL2A1. This action resulted in a blockade of cartilage breakdown and bone loss. The combination of HEM and UC-II also prevented OA progression. These findings provide evidence for using HEM and UC-II for OA therapy.
